# Chitosan Edible Films Enhanced with Pomegranate Peel Extract: Study on Physical, Biological, Thermal, and Barrier Properties

**DOI:** 10.3390/ma14123305

**Published:** 2021-06-15

**Authors:** Nishant Kumar, Pratibha, Anka Trajkovska Petkoska, Ebtihal Khojah, Rokayya Sami, Amina A. M. Al-Mushhin

**Affiliations:** 1 Department of Agricultural and Environmental Sciences, National Institute of Food Technology Entrepreneurship and Management, Sonepat, Kundli 131028, India; 2 Department of Food Business Management and Entrepreneurship Development, National Institute of Food Technology Entrepreneurship and Management, Sonepat, Kundli 131028, India; pratibha91coo@gmail.com; 3 Department of Humanities and Social Sciences, National Institute of Technology, Kurukshetra 136119, India; 4 Faculty of Technology and Technical Sciences, St. Kliment Ohridski University-Bitola, Dimitar Vlahov, 1400 Veles, Republic of North Macedonia; anka.trajkovska@uklo.edu.mk; 5 Department of Food Science and Nutrition, College of Sciences, Taif University, P.O. 11099, Taif 21944, Saudi Arabia; eykhojah@tu.edu.sa; 6 Department of Biology, College of Science and Humanities in Al-Kharj, Prince Sattam Bin Abdulaziz University, Al-Kharj 11942, Saudi Arabia; a.almushhin@psau.edu.sa

**Keywords:** antimicrobial, chitosan, pomegranate peel extract, antioxidant, edible film, properties

## Abstract

In the present study, pomegranate peel extract was used as a reinforcing agent in developing chitosan-based edible film. Different concentrations (0.2 g/mL, 0.4 g/mL, 0.6 g/mL, 0.8 g/mL, and 1.0 g/mL) of pomegranate peel extract were incorporated in chitosan-based edible film. A neat chitosan film was used as a control. This work covers the effect of pomegranate peel extract on the physical, biological, mechanical, thermal, and barrier properties of enriched chitosan-based edible film. The results showed that the thickness (0.142–0.159 mm), tensile strength (32.45–35.23 MPa), moisture (11.23–15.28%), opacity (0.039–0.061%), water (1.32–1.60 g·mm/m^2^), gas barrier properties (93.81–103.45 meq/kg), phenolic content (5.75–32.41 mg/g), and antioxidant activity (23.13–76.54%) of the films increased with increasing volume fraction of pomegranate peel extract. A higher concentration of incorporated pomegranate peel extracts significantly (*p* < 0.05) reduced the thermal stability of the film, along with its transparency, solubility, swelling, and color. This work revealed that the incorporation of a higher portion of pomegranate peel extract in chitosan film holds significant (*p* < 0.05) potential for the increase in biological activities of such films in terms of antioxidant and antimicrobial behavior. The properties of pomegranate peel extract-enriched chitosan films could be an excellent cure for free radicals, whereas they could also inhibit the growth of the foodborne pathogens during the processing and preservation of the food. Further studies are needed for the application of pomegranate peel extract-enriched edible films on food products such as fruits and vegetables in order to extend their storage life and improve the quality and safety of preserved food products.

## 1. Introduction

Recently, the increased awareness of consumers has led to the demand for biodegradable, edible, and active packaging in the food processing sector. Edible coatings and films are eco-friendly materials that help in a reduction in synthetic-based packaging options and deterioration effects on the environment due to their biodegradable nature and other food protection capabilities [1,2,3,4,5]. Edible coatings and films are also known as a modern food protection system. They provide a good protection of food products in terms of a physical, chemical, and thermal barrier [6], as well as prevention of dehydration (moisture loss) and microorganism contamination in food products in a fresh and frozen form [7]. The various types of biodegradable biopolymers such as polysaccharides, proteins, and lipids are usually used in the development of active edible food packaging [8,9]. They act as a carrier of a variety of natural additives and can control the gas and water transmission, as well as mass transfer, between the environment and food products. They can modify the structure of the material via intermolecular interactions [10]. The potential usage of edible materials has been proven in technology for food preservation [11,12,13]. They are an excellent alternative to synthetic packaging that originates from fossil-based polymers such as polyvinyl chloride (PVC), polyethylene (PE), polyvinyl alcohol (PVA), and nylon (PA).

The chitosan biopolymer is commercially used in the food and pharmaceutical sectors due to its suitable physical, chemical and biological properties. It is biodegradable, nontoxic, and biofunctional, and it possesses biocompatible properties [12,13,14,15,16]. Edible films prepared with chitosan-based polymers have an excellent adhesiveness and cohesiveness with a smooth surface of food products [17]. Chitosan is derived from the deacetylation of chitin, which can be found in some fungi and seashells [1,5,8,18]. Moreover, it is a apt material for making edible coatings, primarily because of its nontoxic nature, biocidal activity, and gas barrier properties, useful for prolonging the shelf of food products while maintaining their quality attributes [19,20,21,22,23].

**Figure 1:**
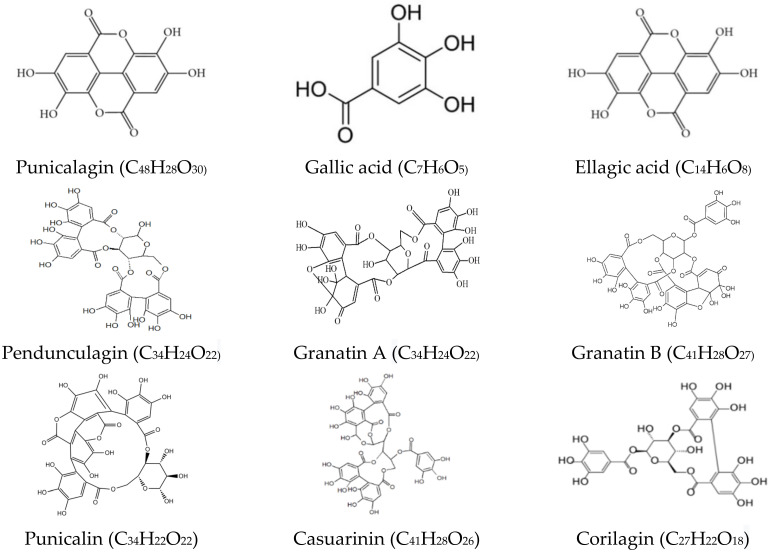
Chemical structure of major phenolic compounds in pomegranate peel waste.

Pomegranate peel produced after the juice extraction of arils contains 48–50% waste material with respect to the total fruit [24]. It is used in the food processing and medical sector as an antioxidative, preservative, and antimicrobial agent because of the presence of hydrolyzable tannins and phenolic compounds [18,25,26,27]. It is a significant source of bioactive compounds such as gallic acid, ellagic acid, punicalagin, quercetin, punicalin, luteolin, kaempferol, glycosides, and pedunculagin [28,29,30]. Figure 1 presents chemical structures of the major bioactive compounds of pomegranate peel. The various studies confirmed that pomegranate peel has higher biological activity (e.g., antioxidant and antimicrobial) compared to other parts of the fruit [30,31,32]. This study aimed to investigate the effect of incorporation of the extract from pomegranate peel as a natural antioxidant and antimicrobial agent on the physical, chemical, mechanical, biological, and thermal properties of chitosan-based edible films.

## 2. Materials and Methods

Low-molecular-weight chitosan, glycerol, methanol, FC reagent, DPPH (2,2-diphenyl-1-picrylhydrazyl), TPTZ (2,4,6-tripyridyl-*s*-triazine), sodium carbonate, gallic acid, quercetin, Muller–Hinton agar, aluminum chloride, and potassium acetate were supplied by Hi-Media and Hi-Tech chemicals (New Delhi, India). The bacterial strain (*E. coli*- NCDC 134) was provided by the National Dairy Research Institute, Karnal (India).

### 2.1. Plant Material

The fresh and fully ripened pomegranate fruits (*Cv. Bhagwa*) were obtained from a farm in Kullu, Himachal Pradesh (India). The procured fruits were clean with deionized water and manually peeled using a sharp knife for freeze-drying of pomegranate peel.

### 2.2. Extraction of Pomegranate Peel (PGP)

The extraction of the freeze-dried pomegranate peel was prepared using the method of Kumar et al. [33]. Pomegranate peel (0.2 g) was dissolved in methanol (10 mL) using ultrasonic-assisted (CUB-5, Citizen, 40 kHz, 220–240 V) treatment at 45 °C for 30 min to recover a higher amount of phenolic extraction. The obtained solution was centrifuged (Sigma, 3–18, KS, Osterode am Harz, Germany) for 10 min at 5 °C at a speed of 8654 rpm to obtain a clear and transparent extract of pomegranate peel. The prepared solution was filtered using Whatman paper No. 11, and the residual methanol solvent was evaporated using a vacuum rotary evaporator at 42 °C to recover phenolic compounds from pomegranate peel powder [34]. The obtained powder of pomegranate peel was used for the preparation of different concentrations (0.2 g/mL, 0.4 g/mL, 0.6 g/mL, 0.8 g/mL, and 1.0 g/mL) of pomegranate peel extract in chitosan-based edible films.

### 2.3. Preparation of Edible Formulations and Casting of the Film

Table 1 shows the formulations of chitosan-based edible coating with different concentrations of pomegranate peel extract. Chitosan solution (2%) was prepared in a 0.5% aqueous solution of citric acid to increase the solubility of chitosan material. The prepared solution was homogenized at a speed of 10,000 rpm for 10 min and continuously stirred for 60 min at room temperature (23 ± 2 °C) using a magnetic stirrer. Then, 5 mL of different concentrations (0.2 g/mL, 0.4 g/mL, 0.6 g/mL, 0.8 g/mL, and 1.0 g/mL) of pomegranate peel extract and glycerol (1%) as a plasticizer were added to the prepared chitosan solutions and stirred again for 60 min at room temperature (23 ± 2 °C). The obtained solution was used for the preparation of the edible film. Next, 100 mL of prepared solution from each formulation enriched with pomegranate peel extract was cast at 40 ± 5 °C for 12 h on 13 cm × 13 cm Teflon glass plates. The obtained dried edible films were conditioned at 25 ± 5 °C for 24 h with 53% relative humidity [35].

### 2.4. Thickness Measurement

The thickness of the prepared chitosan-based edible films was determined using a digital micrometer (293–821, Mitutoyo, Kanagawa, Japan). The average value was reported as the thickness of the edible films and used later for the calculations of barrier and mechanical properties [36].

### 2.5. Moisture Content

The moisture content of the developed edible films was determined using the AOAC gravimetric method with minor modification [37]. First, 10 g of each film specimen was dried at 120 ± 2 °C for 24 h. The results were calculated as a function of the mass loss of the films before and after drying. The moisture content was expressed as a percentage and calculated according to the following formula:

Moisture (%) = (W_2_ − W_3_)/W_1_ (1)

where W_1_ is the weight of the empty petri dish (g), W_2_ is the weight of the petri dish + sample before drying (g), and W_3_ is the weight of the petri dish + sample after drying (g).

### 2.6. Water Solubility

The water solubility of the chitosan edible film was determined according to the method in [36] with minor modifications. The obtained edible films (25 mm × 50 mm) were immersed in 50 mL of distilled water and continuously stirred for 24 h at room temperature (23 ± 2 °C). After this process, the non-solubilized film was taken out, and the solubility of edible films was calculated. The solubility of the edible films was reported as the difference between initial and final weight. The results were expressed as a percentage and calculated according to the following equation:

Water Solubility (%) = (Wi − Wf)/Wi × 100(2)

where Wi is the initial weight of the dried film (g), and Wf is the final weight of the dried (immersed) film (g).

### 2.7. Swelling Property

The swelling degree of the chitosan-based edible films was tested using the conventional method [38] with minor modification. The film samples were cut into samples with dimensions of 1 cm × 2 cm and their weight was measured. The samples were immersed in 25 mL of deionized water at room temperature (23 ± 2 °C), taken out of the deionized water, and wiped well. Lastly, the edible film was weighed in order to measure the swollen capacity, calculated using the following formula:

Swelling degree (%) = (M_1_ − M_2_)/M_2_ × 100 (3)

where M_1_ is the mass of the wet (swollen) film (g), and M_2_ is the mass of the dried film (g).

### 2.8. Optical Properties of Edible Films

#### 2.8.1. Transparency/Opacity

The transparency and the opacity of the developed chitosan-based edible film enriched with pomegranate peel extract were determined according to the methodology in [39,40]. To determine the transparency and opacity of the edible films, each specimen was cut in a rectangular shape and placed in a UV spectrophotometer (Sican 2301, Inkarp Pvt. Ltd., Hyderabad, India) test cell at 600 nm wavelength. A blank (air) was used as a reference for comparative measurement. Results of both transparency and opacity measurements were expressed as a percentage and calculated using the following formulas:

Transparency (%) = Abs_600_/L (4)

Opacity = Abs_600_ × L (5)

where Abs_600_ is the spectrophotometric absorbance value at 600 nm wavelength, and L is the thickness of the edible film in mm.

#### 2.8.2. Color Analysis

The effect of incorporated pomegranate peel extract on the color properties (L*, a*, b*) of chitosan-based films was measured using a handheld chroma meter (CR-400, Konica Minolta, Tokyo, Japan) at room temperature (23 ± 2 °C) [41]. The scale values were recorded in terms of CIE L*, a*, and b* scale color values (L* = 0 (black) to 100 (White), a* = −60 (green) to +60 (red), and b* = −60 (blue) to +60 (yellow)). The chroma meter was calibrated using a calibration standard plate. 

### 2.9. Barrier Properties of Edible Film

#### 2.9.1. Water Barrier Property (WVP)

The water vapor permeability of chitosan-based edible films doped with pomegranate peel extract was measured using a WVP tester (Labthink-Preme w3/030, Jinan, China). The measuring range of the equipment ranged from 0.03 to 1000 g/m^2^/day. Each film specimen was conditioned for 48 h in a desiccator at 23 ± 2 °C and 53% relative humidity before analysis. The dehydrated silica gel filled in the desiccator was placed 9 mm away from the film samples. The test films were cut into round shapes (33 cm^2^). The relative humidity and temperature used in the test were 90% and 38 °C, respectively. The water vapor permeability of the edible films was calculated using the following formula [42,43]:

Water vapor permeability (g·mm/h·m^2^·kPa) = WVTR × L/ΔP (6)

where WVTR is the water vapor transmission rate (g/h·m^2^), L is the thickness of the edible films (mm), and ΔP is the partial water vapor pressure difference (kPa).

#### 2.9.2. Oxygen Barrier Properties 

The oxygen barrier properties of chitosan-based edible films incorporated with pomegranate peel extract were determined according to the method in [44]. For this purpose, 30 mL of soybean oil was placed in a 60 mL jar and covered with edible films. The jar was stored for 10 days at 60 °C. The peroxidase value of the samples was measured by titration with sodium thiosulfate. The oxygen barrier property (peroxidase value) was expressed as meq/kg.

### 2.10. Mechanical Strength

The mechanical strength of chitosan-based edible films incorporated with pomegranate peel extract was determined according to the method followed by Kumar et al. [33]. For this purpose, samples of 2 cm × 8 cm strips were used to determine tensile strength using a Texture analyzer (Stable Microsystems, Goldalming, UK) with crosshead speeds 50 mm and 0.5 mm, respectively. The mean results of tensile strength were expressed in MPa.

Tensile strength = maximum force (N)/thickness (mm) × width (cm)(7)

### 2.11. Thermal Property (DSC Analysis) 

The thermal property of prepared edible films with the incorporation of pomegranate peel extract was determined using differential scanning calorimetry (Netzsch DSC 200 F3 Maia, Selb, Germany) under a nitrogen-conditioned atmosphere [45]. Initially, the instrument was calibrated with indium (156.6 °C—Melting point, ΔH = 28.5 J/g), after which 8–10 mg of edible film samples were used with 20 mL/min flow capacity at −50 to 300 °C at a 10 °C/min heating interval. The glass transition temperature (T_g_) of film samples was determined by taking the first derivative of the thermograms. T_g_ indicates the baseline of the DSC plot in the glass transition, and it is the midpoint of the onset and end-set temperature.

## 3. Phenolic Content and Antioxidant and Antimicrobial Activity of Edible Films

### 3.1. Extraction of Edible Films

For preparation of the edible film extract to determine phenolic content, as well as antioxidant and antimicrobial activity, 5 g of each specimen of the edible film was homogenized with 25 mL of methanol at a mixing rate of 18,000 rpm for 3 min using a homogenizer. The obtained solution was centrifuged (Sigma 3–18KS, Osterode am Harz, Germany) for 10 min at 5000 rpm. The prepared extract was stored in refrigerating conditions for further use [46].

### 3.2. Total Phenolic Content

The total phenolic of prepared edible films was determined using standard Folin–Ciocâlteu (FC) reagent [47]. First, 1 mL of prepared film extract was mixed with 50 mL of distilled water. Then, 1 mL of FC reagent and 15 mL of 20% sodium carbonate were added to the previous mixture. The mixture was incubated for 2 h in a dark place. The absorbance was recorded using a UV spectrophotometer (SICAN 2301) at 765 nm wavelength. The concentration of phenolic compounds in the edible films was determined as mg/g of gallic acid [33,48].

### 3.3. Antioxidant Activity (DPPH Assay) 

The antioxidant activity of prepared edible films was performed using DPPH (2,2-diphenyl-1-picrylhydrazyl) [49]. First, 0.5 mL of edible film extracts were mixed with 1.5 mL of 0.1 mM methanolic DPPH solution. Methanol was used as a reference (control) sample. The absorbance of the samples was recorded at 517 nm wavelength using a spectrophotometer (SICAN 2301). The results of antioxidant activity were expressed as a percentage and calculated using the following equation:

Antioxidant activity (%) = [(A_control_ − on_sample_)/A_control_] × 100 (8)

where A_control_ is the absorbance of the control sample, and A_sample_ is the absorbance of the sample.

### 3.4. Antimicrobial Activity against E. coli

The in vitro antimicrobial activity of the various prepared edible films was investigated using a disc inhibition assay following the method by Zhang et al. [50] against an *E. coli* (NCDC 134) microbial strain. For this purpose, the film samples were cut into a round shape of 10 mm in diameter and sterilized. After that, they were placed on an *E. coli* solid culture medium surface. Lastly, the Petri plate was incubated at 37 °C for 24 h. The antimicrobial activity of the films was measured in terms of the zone of inhibition (mm).

## 4. Statistical Analyses

ANOVA and the Duncan triplicate range test with a *p* < 0.05 level of significance were applied for the analysis of recorded data using SPSS statistical software version (IBM SPSS 24.0) [51]. The graphical presentation of the results was prepared using OriginPro 2019b [52], and the results were expressed as the mean ± standard deviation.

## 5. Results and Discussion

### 5.1. Thickness of Edible Films

**Figure 2:**
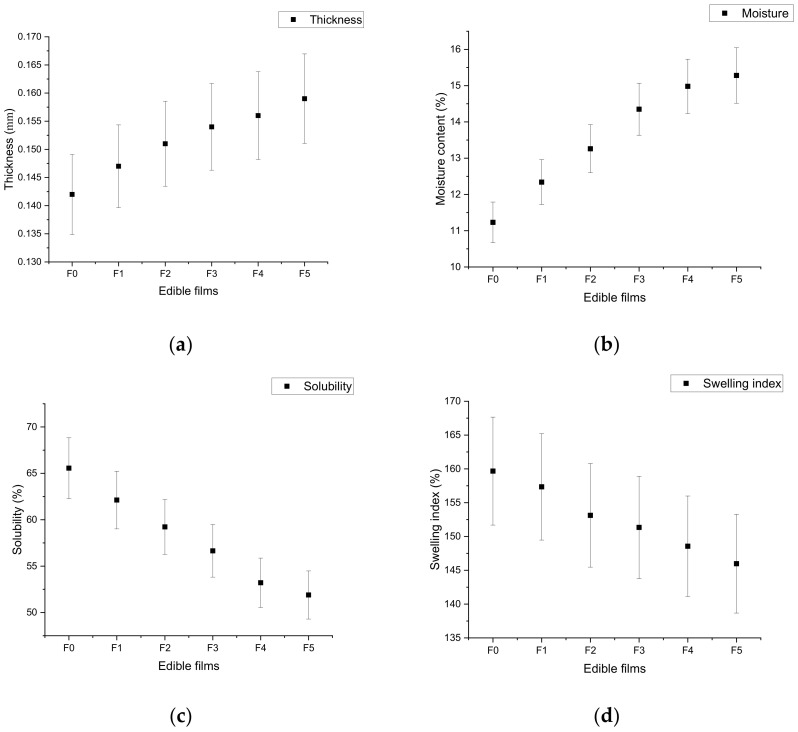
Effect of pomegranate peel extract on thickness (**a**), moisture content (**b**), solubility (**c**), and swelling index (**d**) of chitosan-based edible films. F0 = control (chitosan), F1 = 0.2 g/mL PGP, F2 = 0.4 g/mL PGP, F3 = 0.6 g/mL PGP, F4 = 0.8 g/mL PGP, and F5 = 0.10 g/mL PGP.

Thickness is an important variable affecting the characteristics of the films, such as tensile strength, elongation, and water vapor permeability [53,54]. Thickness directly affects the appearance of the products, along with their barrier properties against water and gas transmission. An increasing rate of edible film thickness helps in reducing the diffusion rate [55]. The thickness of the film material is directly dependent on its preparation methods, such as drying, solvent evaporation time, relative humidity, and dish surface [56]. Park et al. [57] reported the addition of various compounds such as plasticizer, antioxidant, and antimicrobial compounds in the matrix. In this study, the control film samples showed lower thickness (0.142 ± 0.05 mm) compared to other tested samples. The highest thickness was recorded in F5 (0.159 ± 0.43 mm) film samples followed by F4 (0.156 ± 0.28 mm) and F3 (0.154 ± 0.83 mm) film samples (Figure 2a). These results revealed that the thickness of the edible film increased with the increase in concentration of pomegranate peel extract due to intermolecular interactions between the functional groups [58]. The incorporation of pomegranate peel extracts did not significantly (*p* < 0.05) affect the thickness of the chitosan-based films. These results are in line with the previous study by Hoque et al. [59]. They reported that the incorporation of natural antioxidant agents (cinnamon, clove and star anise) did not significantly affect the thickness of the film. Yuan et al. [41] reported that the incorporation of pomegranate peel extract into chitosan-based edible films did not significantly affect the thickness of the developed film. The results of this study strongly agree with those reported by Kumari et al. [60], Nur-Hanani et al. [61], Nur-Hanani et al. [62], and Moghadam et al. [35], who investigated the effect of pomegranate peel extract on the thickness of gluten, gelatin/polyethylene, fish gelatin, and mung bean protein-based films, respectively.

### 5.2. Moisture Content of Edible Films

Figure 2b presents decreasing trends of moisture content of the edible film with an increased amount of pomegranate peel extract. The moisture content of the control (F0) film was determined with a lower value (11.23% ± 0.89%) compared to other tested films. The highest moisture content value was recorded for F5 film samples (15.28% ± 0.45%), followed by F4 (14.98% ± 0.38%) and F3 (13.26% ± 0.58%) film samples. The results showed that the moisture content of the chitosan-based film increased upon increasing the concentration of pomegranate peel extract in the matrix, probably due to molecular interactions and changes in the hygroscopic nature of the chitosan matrix [61]. Similar results were reported by Kumari et al. [60], who found that the moisture content of film was significantly increased (43.53%) after the incorporation of pomegranate peel extract in a gluten-based matrix compared to ordinary film (35.91%). The results of this study agree with a previous study by Augusto et al. [63], who reported that the moisture content of a chitosan- and alginate-based film increased after the incorporation of *Codium tomentosum* seaweed extract.

### 5.3. Water Solubility of Edible Films

Solubility offers many potential benefits for biomaterial devices [64,65,66,67]. The film solubility is directly linked to the structural properties of the matrix and the content of phenolic compounds. In the present study, the solubility of the ordinary chitosan film (F0) was recoded as being higher (65.56% ± 0.32%) compared to the films with pomegranate peel extract. The results of this study indicate that film solubility decreased with the increased concentration of pomegranate peel extract (Figure 2c). The highest significant difference in film solubility was recorded between the control film (F0) and F5 film. These results strongly agree with a previous study by Moghadam et al. [35], who reported that the solubility of the film decreased with the incorporation of pomegranate peel extract. Other studies also showed that incorporation of plant extracts decreased the water solubility of films. Previous researchers reported a decreasing trend of solubility after the incorporation of seaweed [63] and *Ginkgo biloba* [68] extract in alginate and gelatin films, respectively.

### 5.4. Swelling Property of Edible Films

The swelling capacity of a film indicates its biodegradation and applicability on food products, as well as water resistance property [33]. This property predicts the maintenance of quality during the packaging and storage of food products [69]. In some cases, a higher swelling index can be desirable to absorb extra water from the outer surface of foods with high moisture [70]. In fact, as is well known, the degree of swelling of a polymeric material strongly depends on the amount and the nature of intermolecular chain associations [71]. The swelling tendency of the film is a very important factor for fresh-cut fruits with high-moisture surfaces [55]. In this study, the swelling capacity of the film gradually decreased with the presence of pomegranate peel extract in chitosan-based films. The control (F0) film exhibited the highest water resistance capacity compared to other tested films. The chitosan film doped with the highest concentration of (1.0 g/mL) pomegranate peel extract showed the lowest swelling capacity (145.97% ± 0.28%), followed by F4 (148.56% ± 0.34%) and F3 (151.34% ± 0.41%) films (Figure 2d). Overall, the results concluded that the swelling tendency of the chitosan film decreased with the incorporation of pomegranate peel extract. This may have been due to increased crosslinking between the matrix and plant extract [72]. The results of this study are in line with a previous study by Nemazifard et al. [73], who also reported a decreasing trend of the swelling capacity of a cellulose-based film with the incorporation of pomegranate seed extract due to saturation of the matrix with pomegranate seed extract. Mayachiew and Devahastin [72] reported that the swelling degree of a chitosan film was significantly affected by gooseberry extract due to the hydrophobic nature of the extract and its intermolecular interactions with chitosan.

## 6. Optical Properties of Edible Films

Optical properties are important factors for food packaging. The optical parameters of the film directly affect the appearance of the products and their acceptance by consumers [74]. Changes in the optical properties of a film are dependent mainly on the incorporated bioactive compounds. Various studies have reported that the incorporation of plant extract could affect the optical (transparency, opacity, and color) properties of films by increasing yellowness and redness values, as well as decreasing lightness [58].

### 6.1. Transparency/Opacity of Edible Films

**Figure 3:**
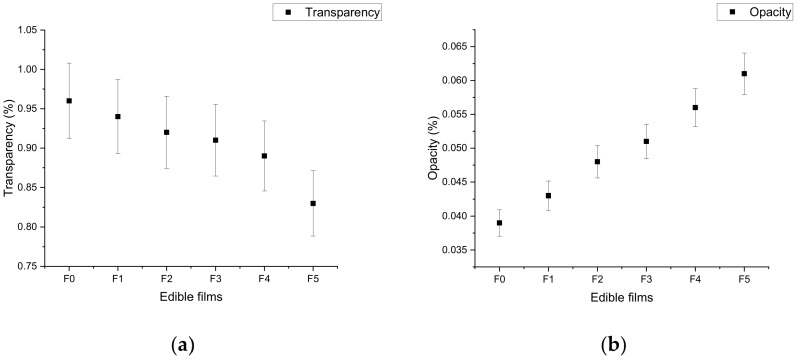
Effect of pomegranate peel extract on the transparency (**a**)/opacity(**b**) of chitosan-based edible films. F0 = control (chitosan), F1 = 0.02 g/mL PGP, F2 = 0.4 g/mL PGP, F3 = 0.6 g/mL PGP, F4 = 0.8 g/mL PGP, and F5 = 0.10 g/mL PGP.

Transparency or opacity is an important physical property of packaging films, describing the see-through property or prevention of light transmission. A higher transmittance value of the film denotes better transparency, due to the fact that more visible light (660 nm) is able to pass through the film [75]. The results of transparency and opacity of the developed films are expressed in Figure 3a,b. The transparency and opacity of the film samples showed decreasing and increasing trends, respectively, with the incorporation of pomegranate peel extract. Increasing the concentration of pomegranate peel significantly (*p* < 0.05) affected the transparency and opacity of the film samples. The control film sample (F0) showed higher transparency (0.96% ± 0.46%) with 0.039% ± 0.81% opacity. The chitosan film with the highest incorporated concentration of pomegranate peel extract (F5) showed the lowest transparency (0.83% ± 0.19%) with maximum opacity (0.061% ± 0.58%) due to the presence of phenolic fragments. The results clearly show that the inclusion of pomegranate peel extract decreased the transparency and increased the opacity of chitosan films. The results are in accordance with previous study by Qin et al. [40], who reported that the transparency of a chitosan-based film with incorporated pomegranate peel extract showed a reduction due to the availability of hydrolyzable tannin compounds of pomegranate peel, which altered the pores of the inner structure of the film [36]. In this context, Gómez-Estaca et al. [76] reported a significant (*p* < 0.05) decreasing trend of the transparency and increasing trend of the opacity of a gelatin film after the incorporation of plant (rosemary and oregano) extracts.

### 6.2. Color of Edible Films

**Figure 4:**
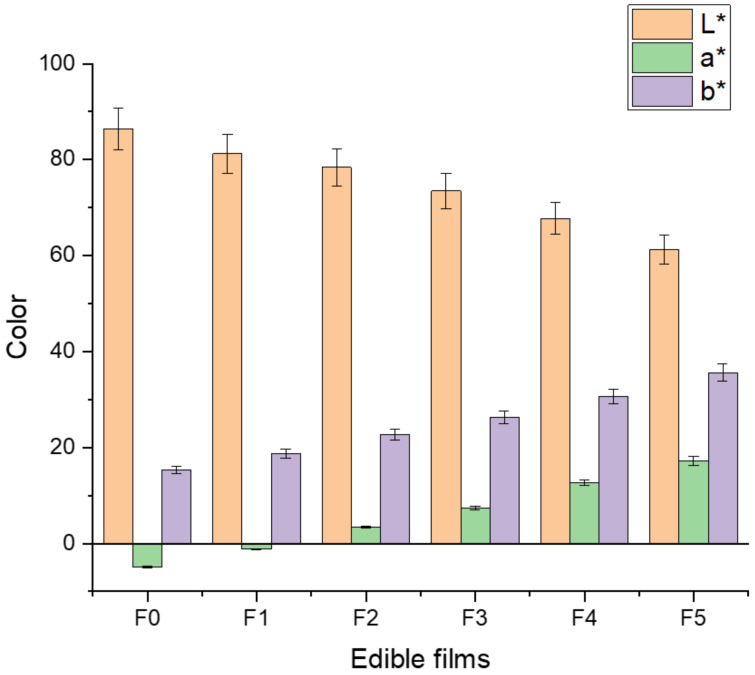
Effect of pomegranate peel extract on color (L*, a*, and b*) of chitosan-based edible films. F0 = control (chitosan), F1 = 0.02 g/mL PGP, F2 = 0.4 g/mL PGP, F3 = 0.6 g/mL PGP, F4 = 0.8 g/mL PGP, and F5 = 0.10 g/mL PGP.

Three important aspects of food acceptance are color, flavor, and texture. The color characteristics of edible films are important physical properties that affect the appearance of the products. However, an increased brightness of the edible film denotes its better quality [77]. The data obtained for the film samples in terms of color parameters are expressed in Figure 4. The present study revealed that the darkness, redness, and yellowness of the film samples increased with increasing concentration of pomegranate peel extract in the film due to the presence of antioxidants and anthocyanin pigments. The control sample film (F0) showed a higher lightness with higher transparency compared to other films. The lightness of the films decreased with increasing concentration of pomegranate peel extract. The darkest color was recorded in the F5 film compared to other film samples. The results of this study showed that the incorporation of pomegranate peel extract significantly (*p* < 0.05) affected the optical (color) properties of the film sample. It has been reported that the color of a film is directly affected by the concentration and type of extract due to the presence of antioxidant agents [70]. In agreement with our results, López-Mata et al. [78] and Kanatt et al. [79] reported that the lightness of chitosan films decreased with an increase in yellow and red color after the incorporation of pomegranate peel extract. Similar findings were also reported by Moghadam et al. [35] and Yuan et al. [41].

## 7. Barrier Properties of Edible Films

### 7.1. Water Barrier Property of Edible Films

**Figure 5:**
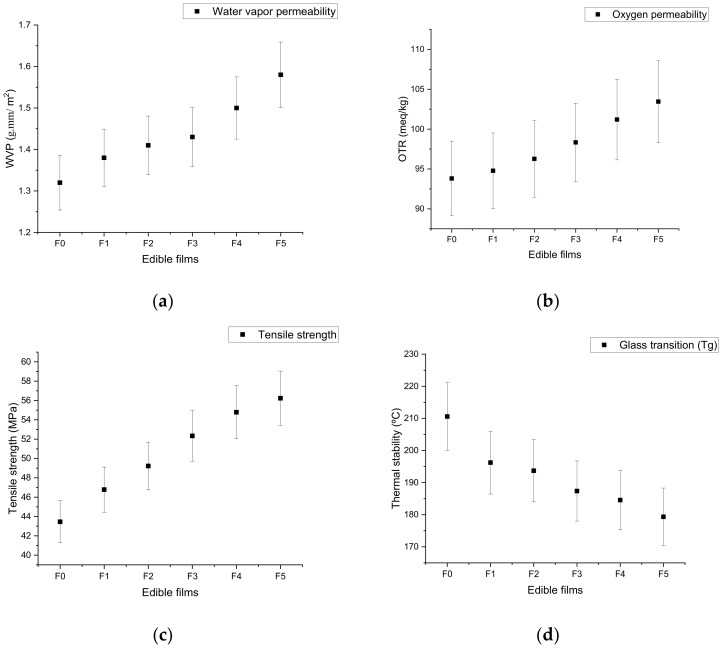
Effect of pomegranate peel extract on water vapor permeability (**a**), oxygen permeability (**b**), tensile strength (**c**), and thermal stability (**d**) of chitosan-based edible films. F0 = control (chitosan), F1 = 0.2 g/mL PGP, F2 = 0.4 g/mL PGP, F3 = 0.6 g/mL PGP, F4 = 0.8 g/mL PGP, and F5 = 0.10 g/mL PGP.

Water vapor permeability (WVP) is an important parameter in the food packaging sector, which indicates the barrier properties toward water transmission. The WVP of a film plays an important role in extending the shelf-life of food products by inducing a decrease in moisture transfer [80]. The WVP of an edible film can be determined on the basis of the mass transfer mechanisms of different components between the food and edible packaging environment, as well as due to interactions between edible materials and food components (e.g., polymer–polyphenol interactions) [81]. The thickness and uniformity of an edible film are important parameters underlying the reliability of the determination of barrier properties such as water vapor permeability [82]. Figure 5a presents the WVP of all samples studied in this work. The WVP of edible films enriched with pomegranate peel extract ranged from 1.38 ± 0.47 to 1.60 ± 0.51 g·mm/m^2^. The water vapor permeability of the chitosan-based films incorporated with pomegranate peel extract slightly increased with respect to the control film (*p* > 0.05) due to the presence of phenolic fractions in the pomegranate peel extract. These phenolic compounds of pomegranate peel formed alternative pathways and cracks in the matrix chemical bonds [83]. Nur-Hanani et al. [62] reported that the addition of pomegranate peel extract to a gelatin-based film showed an increasing trend of water vapor permeability due to weak molecular interactions between the matrix and extract. Covalent and hydrogen bonding interactions between the phenolic compounds of tea extract and chitosan decreased the film’s affinity toward water molecules [84]. Qin et al. [40] identified the interactions between the phenolic compounds of pomegranate rind and the chitosan matrix using Fourier-transform infrared spectroscopy. Our results are in good agreement with the previous studies reported by Moghadam et al. [35] and Yuan et al. [42].

### 7.2. Oxygen Barrier Properties of Edible Films

The oxygen barrier property of a film is represented by its peroxidase value, which indicates the total amount of oxidized substances. The oxygen barrier properties of a film enhance the shelf-life and improve the quality of food products due to the controlled degradation of phenolic compounds and enzymatic reactions [85]. The results pertaining to the oxygen barrier properties of the developed edible films are expressed in Figure 5b. It is apparent that the developed chitosan film without pomegranate peel extract exhibited a significantly (*p* < 0.05) lower value of oxidized oxygen in comparison to the other films. The results show that the oxidation preventability of the F1 film was apparently greater (93.81 ± 0.27 meq/kg) compared to other film samples enriched with pomegranate peel extract. Upon increasing the amount of pomegranate peel extract in the chitosan films, the oxidation preventability decreased due to the lower rate of molecular interactions between chitosan and the pomegranate peel extract, as well as the higher mobility of polymer chains [86]. These results are in line with previous studies by Valdés et al. [83] and Giménez et al. [87], who reported that the oxidation preventability of gelatin- and agar-based films decreased with the incorporation of pomegranate peel and green tea extract, respectively.

## 8. Mechanical Strength of Edible Films

Tensile strength is the main indicator of film strength and flexibility. It can be ascribed to the cohesion between the matrices of the film’s polymer chains. The mechanical strength of the chitosan films is expressed in Figure 5c. The tensile strength of chitosan films with incorporated pomegranate peel extract ranged from 32.45 ± 0.98 to 35.23 ± 0.61 MPa. The results show that the incorporation of pomegranate peel extract had a slight effect on the tensile strength of the films. Increasing the concentration of pomegranate peel slightly increased the mechanical strength, probably due to the increase in free volume, flexibility, and molecular mobility of chitosan matrix chains [35]. The addition of pomegranate peel extract provided rigidity in the chitosan film due to the presence of natural and phenolic fractions. Natural compounds of pomegranate peel act as a natural filler to reinforce the chitosan matrix [27]. These findings are similar to a previous study by Moghadam et al. [35], who reported that the addition of pomegranate peel extract significantly increased the tensile strength of the film due to the interaction between the matrix and phenolic fractions. Siripatrawan and Harte [85] reported that the incorporation of green tea extract significantly enhanced the mechanical strength of a chitosan-based film due to the interaction between the matrix and natural bioactive compounds. Many researchers have reported that the incorporation of gallic acid significantly increased the mechanical strength of edible films, along with phenolic and antioxidant activity [88].

## 9. Thermal Property (DSC Analysis) of Edible Films

A thermal stability test (DSC analysis) is usually performed to study the degradation characteristics of films [89,90]. The glass transition temperatures of the film samples are graphically reported in Figure 5d. The results of this study showed that the glass transition temperature of pure chitosan film (F1) was highest at 210.57 ± 1.32 °C compared to other film samples. A similar trend of glass transition temperature for pure chitosan-based film was reported by Sakurai et al. [91]. Addition of the highest concentration (1.0 g/mL) of pomegranate peel extract led to a higher thermal degradation (179.34 ± 0.92 °C) of chitosan films. 

These results also indicated that the glass transition (T_g_) temperature of film samples decreased upon increasing the concentration of pomegranate peel extract. Kumar et al. [33] reported that the incorporation of pomegranate peel extract decreased the thermal stability of chitosan-based film. Furthermore, Menzel et al. [92] also reported that the addition of natural antioxidants reduced the thermal stability of a starch-based film due to depolymerization and dehydration of the matrix. The results of this study are also in a good agreement with previous studies by Peng et al. [93] and Kaya et al. [94], who reported that the incorporation of natural plant extracts of tea and *Pistacia terebinthus* reduced the thermal stability and phase inversion temperature of a chitosan-based edible film by decreasing the crystalline property of the matrix.

## 10. Phenolic Content and Antioxidant and Antimicrobial Activity of Edible Films

### 10.1. Total Phenolic Content of Edible Film

**Figure 6:**
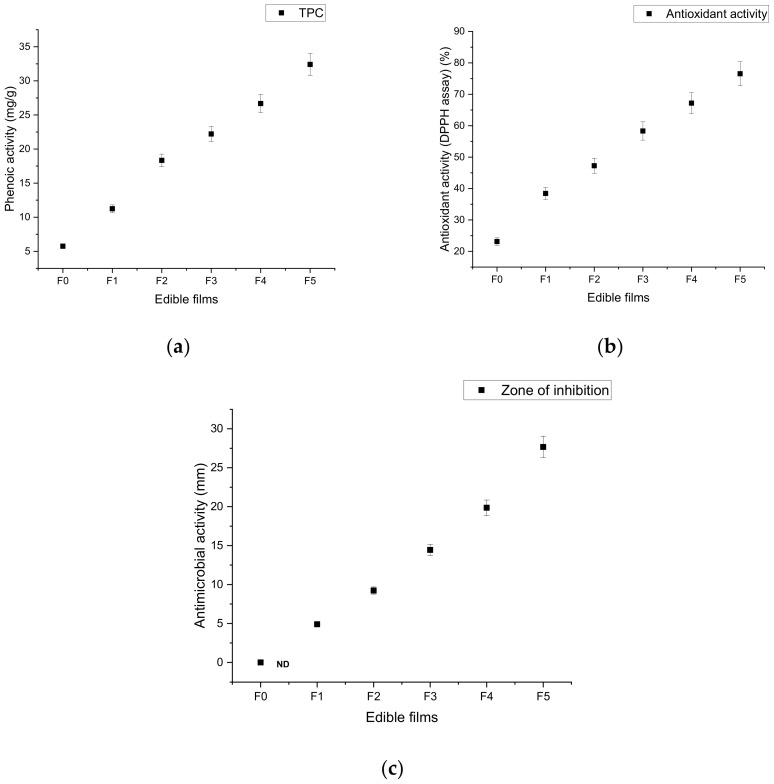
Effect of pomegranate peel extract on total phenolic content (**a**), antioxidant activity (**b**), and antimicrobial activity (**c**) of chitosan-based edible films. F0 = control (chitosan), F1 = 0.2 g/mL PGP, F2 = 0.4 g/mL PGP, F3 = 0.6 g/mL PGP, F4 = 0.8 g/mL PGP, and F5 = 0.10 g/mL PGP.

Phenolic compounds are naturally present in fruits and vegetables in the form of secondary metabolites. These phenolic compounds possess free-radical scavenging activity during oxidative stress [95]. In this study, the addition of different concentrations of pomegranate peel extract revealed different phenolic content in the edible films: 0.2 g/mL (11.25 ± 0.29 mg/g), 0.4 g/mL (18.34 ± 0.34 mg/g), 0.6 g/mL (22.21 ± 0.18 mg/g), 0.8 g/mL (26.68 ± 0.31 mg/g), and 1.0 g/mL (32.41 ± 0.45 mg/g) (Figure 6a). In this study, the control film (F0) showed the lowest (5.75 ± 0.41 mg/g) phenolic content in comparison with other tested films. Increasing the concentration of pomegranate peel extract significantly enhanced the phenolic activity of chitosan-based edible films due to the presence of polyphenolic compounds (gallic acid, ellagic acid, punicalagin, quercetin, catechin, etc.) in pomegranate peel [25,96]. These results are in a good agreement with previous studies by Qin et al. [40] and Mabrouk et al. [97], who reported that the addition of pomegranate peel extract increased the phenolic activity of chitosan- and pectin-based films. In this context, Fan et al. [98] showed a 13-fold increase in the phenolic activity (from 3.21 ± 0.12 to 43.07 ± 0.07 mg/g) of a chitosan-based film with the incorporation of 3% pomegranate peel extract.

### 10.2. Antioxidant Activity (DPPH) of Edible Film

The elimination of free radicals is mostly required to reduce the oxidative stress of fruits and vegetables [99]. Oxidative stress results from damage to molecular species including proteins, lipids, and nucleic acids, due to imbalance between antioxidants and free radicals [100]. Plants are a potent source of natural antioxidant compounds. Natural antioxidant compounds possess better properties as compared to synthetic ones, and they are considered the most important nutraceuticals for eliminating various diseases through free-radical scavenging [101]. Pomegranate peel is an excellent source of natural antioxidant agents such as phenolics, flavonoids, and hydrolyzable tannins [102,103]. Due to its excellent biological properties, pomegranate peel can be used in the food packaging sector to minimize lipid peroxidation. In the present study, the antioxidant activity of chitosan films increased with increasing concentration of pomegranate peel extract (Figure 6b) due to the biological functionality of pomegranate peel with the chitosan matrix. The lowest antioxidant activity (23.13% ± 0.21%) was recorded for the pure chitosan-based film as compared to pomegranate-enriched films. The highest antioxidant capacity (76.54% ± 0.34%) was shown by the chitosan film doped with 1.0 g/mL concentrated pomegranate peel extract (F5), followed by the F4 and F3 films. Other studies have reported that pomegranate peel extract enhanced the antioxidant capacity of chitosan-based edible films [40]. Similar data were reported by Yuan et al. [41], Yuan et al. [42], Kannat et al. [79], and Fan et al. [98], who found that the incorporation of pomegranate peel extract improved the antioxidant activity of chitosan- and PVA-based films.

### 10.3. Antimicrobial Activity of Edible Films against E.coli

The natural antioxidant agents of pomegranate peel act as excellent inhibitors, controlling and reducing the growth of microorganisms. Natural antioxidant compounds are very useful in the food processing industries to retard and control the spoilage of food products [104]. Various studies have confirmed the antimicrobial activities of pomegranate peel against Gram-positive and Gram-negative microorganisms [105]. Pomegranate peel also has the ability to retard and inhibit pathogens. After the incorporation of pomegranate peel extract into the chitosan-based material films, their inhibitory activity toward Gram-positive and Gram-negative microbes was significant [41]. The results of the present investigation are shown in Figure 6c. The results present that the antimicrobial activity of edible films against *E. coli* was significantly (*p* < 0.05) increased with increasing concentration of pomegranate peel extract in the chitosan films. The extract-free chitosan-based edible film (F1) did not affect the growth of *E. coli.* The maximum inhibition activity against *E. coli* was recorded by the F5 film (27.67 ± 0.13 mm) due to the highest phenolic content. The antimicrobial properties of the pure chitosan-based film were negligible due to its insoluble form. The incorporation of natural antioxidants into the edible films inhibited the growth of Gram-negative bacteria due to the diffusion of antimicrobial agents into the cell wall. These results are supported by the studies of Emam-Djomeh et al. [36], Yuan et al. [41], Nur-Hanani et al. [62], and Mabrouk et al. [97], who reported the incorporation of pomegranate peel extract into chitosan-, sodium caseinate-, gelatin-, and pectin-based films exhibited antimicrobial activity against an *E. coli* strain.

## 11. Conclusions

This study reported the development and characterization of chitosan-based edible films containing different loading portions of pomegranate peel extract as an antimicrobial, antioxidant, and reinforcing agent. It was found that the incorporation of pomegranate peel extract into chitosan films significantly increased the phenolic content, antioxidant activity, and mechanical properties in a concentration-dependent manner. All chitosan films enriched with pomegranate peel exhibited significant antibacterial activity against a food-borne pathogen (*E. coli*). Further research is needed to improve the barrier and thermal properties of chitosan edible films enriched with pomegranate peel extract without affecting their physical and biological properties.

## Figures and Tables

**Table 1:** Formulations of chitosan-based edible coating enriched with PGP.

Film Formulations	Chitosan (mL) (*v*/*v*)	PGP-5% (*v*/*v*)
F0	100	0
F1	95	0.2 g/mL
F2	95	0.4 g/mL
F3	95	0.6 g/mL
F4	95	0.8 g/mL
F5	95	1.0 g/mL

F0 = control (chitosan), F1 = 0.2 g/mL PGP, F2 = 0.4 g/mL PGP, F3 = 0.6 g/mL PGP, F4 = 0.8 g/mL PGP, and F5 = 0.10 g/mL PGP (PGP = pomegranate peel extract).

## Data Availability

Available upon request from the corresponding author.
